# Solid Particle Number (SPN) Portable Emissions Measurement Systems (PEMS) in the European Legislation: A Review

**DOI:** 10.3390/ijerph16234819

**Published:** 2019-11-30

**Authors:** Barouch Giechaskiel, Pierre Bonnel, Adolfo Perujo, Panagiota Dilara

**Affiliations:** 1 European Commission, Joint Research Centre, 21027 Ispra, Italy; pierre.bonnel@ec.europa.eu (P.B.); Adolfo.Perujo@ec.europa.eu (A.P.); 2 European Commission, DG-GROW, 1040 Brussels, Belgium; panagiota.dilara@ec.europa.eu

**Keywords:** air pollution, vehicle emissions, on-road testing, portable emissions measurement systems (PEMS), real-driving emissions (RDE), solid particle number, particle measurement programme (PMP), sub-23 nm

## Abstract

Portable emissions measurement systems (PEMS) for gaseous pollutants were firstly introduced in the United States regulation to check the in-use compliance of heavy-duty engines, avoiding the high costs of removing the engine and testing it on a dynamometer in the laboratory. In Europe, the in-service conformity of heavy-duty engines has been checked with PEMS for gaseous pollutants since 2014. To strengthen emissions regulations with a view to minimise the differences between on-road and laboratory emission levels in some cases, PEMS testing, including solid particle number (SPN), was introduced for the type-approval of light-duty vehicles in Europe in 2017 and for in-service conformity in 2019. SPN-PEMS for heavy-duty engines will be introduced in 2021. This paper gives an overview of the studies for SPN-PEMS from early 2013 with the first prototypes until the latest testing and improvements in 2019. The first prototype diffusion charger (DC) based systems had high differences from the reference laboratory systems at the first light-duty vehicles campaign. Tightening of the technical requirements and improvements from the instrument manufacturers resulted in differences of around 50%. Similar differences were found in an inter-laboratory comparison exercise with the best performing DC- and CPC- (condensation particle counter) based system. The heavy-duty evaluation phase at a single lab and later at various European laboratories revealed higher differences due to the small size of the urea generated particles and their high charge at elevated temperatures. This issue, along with robustness at low ambient temperatures, was addressed by the instrument manufacturers bringing the measurement uncertainty to the 50% levels. This measurement uncertainty needs to be considered at the on-road emission results measured with PEMS.

## 1. Introduction

Air pollution from vehicles is important in cities [1,2,3], with road traffic contributing around 11–25% to the particulate matter (PM) concentrations in Europe [4,5]. The contribution is higher in Asia [4,6]. For other metrics, such as black carbon [7] and particle number, the contribution can exceed 90% in busy roads and tunnels [8].

PM mass emissions limits were first set in 1992 for diesel vehicles [9]. The procedure requires testing of light-duty vehicles on chassis dynamometers following specific driving cycles. The whole exhaust gas is diluted in a dilution tunnel with constant volume sampling (CVS). A small part of the diluted exhaust gas is sampled continuously during the test in bags and analysed at the end of the test for gaseous pollutants. Another sample passes through a pre-weighted and conditioned filter. The increase of the filter weight determines the PM mass emissions. Heavy-duty engines are tested on dynamometers with either a full dilution tunnel or a proportional partial flow diluter [10] with a similar approach to the measurement of PM mass emission, as in the case of light-duty vehicles.

The introduction of diesel particulate filters (DPFs) at light-duty vehicles in the early 2000s resulted in very low PM mass emissions, close to the detection limit of the gravimetric method. The task of the particle measurement programme (PMP) was to find a new methodology with a better limit of detection suitable for DPF-equipped vehicles, good repeatability, and low investment costs. The final decided method was based upon counting solid (non-volatile) particles >23 nm [11]. A minimum diameter of 23 nm size was selected in order to exclude volatile nucleation mode particles that could result in repeatability and reproducibility levels not acceptable for legislative purposes [12,13] (more details in Appendix A).

Solid particle number (SPN) limits were introduced in 2011 (Euro 5b) in the European Union (EU) for diesel light-duty vehicles with a limit of 6 × 10^11^ p/km. After further evaluations of the methodology for heavy-duty engines, an SPN limit was introduced in 2013 for heavy-duty diesel engines and in 2014 for positive ignition heavy-duty engines (6 × 10^11^ p/kWh weighted 14% for the cold start cycle and 86% for the hot start one). In 2017, non-road engines (19–560 kW), inland waterway vessels (>300 kW), and rail traction engines were included with the limit of 1 × 10^12^ p/kWh. Light-duty gasoline direct injection vehicles were covered in 2014, initially with a higher limit of 6 × 10^12^ p/km upon request of the manufacturer. Since 2017, the same limit as diesel engines has applied. For passenger cars, the limits also have to be respected on the road with the real-driving emissions (RDE) regulation. The regulatory framework was described elsewhere [14]. 

Most laboratories cannot simulate some on-road situations, for example, large road gradients, strong accelerations, and variations in altitude and ambient temperature [15]. In some cases, on-road emissions are much higher than the laboratory ones [16,17,18,19,20,21,22,23], although emissions regulations prescribe that emission limits need to be respected under normal conditions of use [24]. On-road measurements are conducted with portable emissions measurement systems (PEMS) [25,26] and can provide emissions data under a wide range of engine operating conditions [17]. PEMS were introduced in the United States regulation in 2005 for in-use testing of heavy-duty engines. Europe introduced them in 2014 for heavy-duty engines (Regulation (EU) No 582/2011) and in 2017 for light-duty vehicles (Regulation (EU) 2016/427). Today PEMS are robust and reliable tools for testing on the road, with their measurement performance and uncertainty well defined [27].

PEMS for solid particle number (SPN) emissions became available only recently [18]. For this reason, comparisons of on-road and laboratory vehicle emissions are quite limited [18,28]. The first evaluations started in 2013, and in 2017, SPN-PEMS were introduced in the European light-duty legislation (Regulation (EU) 2017/1154). For heavy-duty vehicles, SPN-PEMS measurements were recently introduced in European legislation and will be required by 2021 (Regulation (EU) 2019/1939). In general, the more relaxed technical specifications of SPN-PEMS result in higher measurement uncertainty than measurements in the laboratory (more details in Appendix A). PEMS measure undiluted exhaust gas directly from the tailpipe, while the laboratory systems diluted exhaust from the dilution tunnel. The particle concentrations might not be identical due to coagulation, thermophoresis, and diffusion [29,30] or the different sampling conditions [31,32]. For the above reasons, a 50% and 63% measurement uncertainty is applicable to SPN on-board measurements for light and heavy-duty applications, respectively.

The objective of this paper is to give a historical overview of the introduction of PEMS for particle number in the European regulation and summarise all studies conducted on the topic for the determination of their measurement uncertainty.

## 2. PEMS Development 

In order for a light-duty vehicle or heavy-duty engine to enter the market, the applicable limits of various pollutants have to be fulfilled at type-approval and during in-service conformity, i.e., on vehicles already in the market. These tests will be complemented by the requirement to test vehicles for market surveillance starting in mid-2020. The difficulty of testing vehicles in the market for heavy-duty vehicles is clear: One would need to remove the engine from an in-use vehicle, and therefore, there was the pressing need to be able to test the emissions of the whole vehicle. 

### 2.1. PEMS Origins

The first on-board measurement systems were designed and tested in the 1990s [33,34]. The on-board systems may range from simple sensors with the respective auxiliaries up to heavy and large instruments. In this paper, the terminology used in the European regulation will be followed. Thus, a PEMS is composed from the following components [26]:

- Analysers to determine the concentration of pollutants in the exhaust gas.
- One or multiple instruments or sensors to measure or determine the exhaust mass flow.
- A Global Positioning System (GPS) to obtain the position, altitude, and speed of the vehicle.
- If applicable, sensors and other appliances not being part of the vehicle, for example, to measure ambient temperature, relative humidity, air pressure, and vehicle speed.
- An energy source independent of the vehicle to power the PEMS for light-duty vehicles. In the case of heavy-duty vehicles, the electrical power for the PEMS system may be supplied by the internal electrical system of the vehicle as long as the power demand for the test equipment does not increase the output from the engine by more than 1% of its maximum power.

Other terms such as sensors, mini PEMS, small emission measurement systems (SEMS), on-board monitors (OBM) usually lack some of the above-mentioned components (e.g., the energy source or the flow meter, etc.) and will only be shortly discussed at the end of the review.

### 2.2. PEMS in the Unites States

Beginning in 2001, the United States Environmental Protection Agency (EPA), California Air Resource Board (CARB), and heavy-duty engine manufacturers collaborated to develop an in-use testing and compliance program based on performing the not-to-exceed (NTE) certification test. The method, which was agreed in 2003, requires that the emissions of a pollutant have to be calculated from real-time signals for a period of time where the engine operating conditions fall into a defined high speed and load area of the engine map for a minimum of 30 s, called an “NTE event”. The in-use test is conducted with an on-board PEMS during real in-use operation, avoiding the high costs of removing the engine from a vehicle in normal use and testing it on a dynamometer in the laboratory. In June 2005, EPA adopted the heavy-duty in-use testing regulation, in which it mandated the measurement of gaseous pollutants for 2007 and later engines [35]. PM mass measurements were mandated later (2011 and later engines) due to lack of suitable equipment and the need for separate evaluation of their measurement uncertainty [36].

### 2.3. PEMS in Europe (Heavy-Duty)

In Europe, PEMS evaluation for in-service conformity (ISC) started in 2004. After the first positive conclusions, the Commission launched a pilot program for heavy-duty engines in 2006 through a technical expert group of the European manufacturers, the member states, and the type approval authorities. The main conclusion was that the performance (accuracy, repeatability) of the analysers and exhaust mass flow meters was satisfactory for the purposes of ISC [37]. The European PEMS consortium proposed an alternative method (moving averaging window) because the United States NTE methodology was not suitable for the European heavy-duty vehicles since only a limited share (max. 20%) of the test data could be used to assess the engine conformity. 

According to the moving window method, the real-time signals of a pollutant are used to calculate the emissions over a period of time (window) in which the engine work (or CO_2_ emissions) matches the engine type approval values on the applicable engine transient cycle. For a given on-road test, many windows of different durations are calculated, depending on the operating conditions of the vehicle [25]. The tool developed by the Joint Research Centre (JRC) of the Commission to post-process the data based on the moving average windows is called “EMROAD”. 

Gaseous PEMS were introduced in the European ISC Euro VI heavy-duty regulation in 2014 (Regulation (EU) 582/2011). The first test with PEMS has to be conducted on a demonstration vehicle during the type approval. The result should not exceed the (Euro VI) limit considering a conformity factor (1.5 for gaseous pollutants) that takes into account the PEMS measurement uncertainty. 

The first European evaluation study did not include any PM mass PEMS (PM-PEMS). The PM-PEMS evaluation started in 2008 with participation of the Commission, PEMS manufacturers, and the European association of heavy-duty engine manufacturers (ACEA). The main requirement for PM-PEMS was a good correlation with the (regulated) gravimetric (filter)-based mass method [38]. Most systems used a real-time signal (surface area, black carbon) in combination with an integrated PM mass determined by filter loading. The results of the PM-PEMS project, which lasted approximately two years, were satisfactory [39,40,41]. It was concluded that PM-PEMS measurement instruments were ready to be introduced in the regulation. The analysis used for gaseous pollutants could also be used for particles [42]. In 2016 though, a lot of progress had been made in the light-duty vehicles real-driving emissions (RDE) regulation (see next section), and it was decided to evaluate SPN-PEMS also for the heavy-duty sector. The solid particle number (SPN) method, due to its superior sensitivity, was already considered as an alternative in 2009, although there were no available instruments at that time [39]. The main evaluation started in 2016 and finished in 2017 with some further testing in 2018. SPN-PEMS will be introduced with the Euro VI step E regulation with conformity factor 1.63, based on the heavy-duty engines studies (see paragraph 3.2.4. for more details) (Regulation (EU) 2019/1939). 

Regarding non-road mobile machinery (NRMM), in-service monitoring (no conformity factor) is applicable to 56–560 kW engines (Regulation 2017/655) with PEMS but only for gaseous pollutants.

### 2.4. PEMS in Europe (Light-Duty)

Air quality data showed improvements in NO_2_ concentration lower than expected, and JRC started exploring the use of PEMS to monitor emissions of light-duty vehicles in 2006 [21]. The gap between official laboratory results [16] and the actual on-road emissions, especially for NO_x_, led to a decision to study a new method to measure pollutants during real use of the vehicle. This was based on Regulation 715/2007 that stated that real-world emissions should correspond to the levels measured during type approval. In 2011 a technical working group on real-driving emissions (RDE) was set up and led to the introduction of several pieces of legislation. Commission Regulation (EU) 2016/427 (first regulatory act of the RDE regulation) introduced for the first time an on-road testing with PEMS to complement the laboratory Type I test for the type approval of light-duty vehicles in the EU. It also included the RDE test protocol, instrumentation for gaseous emissions, and data evaluation rules. Subsequently, Commission Regulation (EU) 2016/646 introduced the NTE concept, which requires each test to remain below the emission limit multiplied by a so-called conformity factor (CF) that takes into account the measurement uncertainty of the PEMS. A temporary CF for NO_x_ was set at 2.1 and could be used upon the request of the manufacturer in Europe from September 2017/2019 (new models/all new vehicles). The final CF for NO_x_ for January 2020/2021 (new models/all new vehicles) was originally set at 1.5. Both regulations were consolidated in the WLTP Regulation (EU) 2017/1151.

Regulation (EU) 459/2012 required attention to the particle emissions of positive ignition vehicles under real driving conditions. The Commission had to develop and introduce corresponding measurement procedures within three years of the Euro 6 introduction (i.e., until 2017). The interest for developing a procedure for on the road testing using portable emission measurement systems (PEMS) for SPN emissions (SPN-PEMS) of light-duty vehicles was announced in November 2012, the kick-off meeting took place in April 2013, and the work lasted until the end of 2015. Regulation (EU) 2017/1154 (the third part of the RDE) introduced a conformity factor of 1.5 for the SPN RDE tests (September 2017 for new models). Regulation 2018/1832 (the fourth part of the RDE) introduced on-road emissions testing as part of in-service conformity checks from 2019 and slightly lowered the conformity factor for NO_x_ from 1.5 to 1.43 in 2018 following a review [27]. Regulation (EU) 2018/858 introduced, among others, testing with PEMS for market surveillance purposes from 2020 in the EU. In late 2018, the General Court in Europe ruled against the Commission for its use of conformity factors for NO_x_. The Commission in 2019 appealed the decision and made a proposal to move the conformity factors in the main Regulation (EC) 715/2007.

### 2.5. PEMS in Asia

In China, gaseous PEMS will be introduced for heavy-duty type approval and in-service conformity with China VIa (2020), while SPN will be introduced with China VIb (2023). Light-duty China 6b enforces compliance with RDE, starting 2023 (nationwide). The definition of RDE and the boundary conditions (e.g., altitude, inclusion or not of cold start) are still under discussion. A monitoring phase of RDE starts with 6a, and the conformity factors will be decided later. The regulations apply to all vehicles, in contrast to Europe, where SPN regulations apply to gasoline direct injection vehicles only. 

The Indian Bharat Stage 6 norms are applicable starting in April 2020, although full implementation to include RDE will start in April 2023. The RDE details are still under discussion in order to better reflect the local driving conditions, which are very different from those in Europe.

In Japan, heavy-duty in-service conformity with PEMS is under discussion, with the earliest introduction in 2022. Japan plans to introduce RDE regulation for certain diesel vehicles in 2022. SPN is not regulated under the laboratory (chassis-dyno) conditions and, consequently, on the road. 

In South Korea, for heavy-duty in-service conformity, gaseous PEMS testing was required with Euro VI from 2016. For light-duty, RDE will be introduced in 2020 (including SPN).

### 2.6. Global Technical Regulation

A Global Technical Regulation (GTR) on RDE is being prepared under the auspices of the United Nations (UN). The text being prepared for adoption in June 2020 by the UN working party on pollution and energy (GRPE) describes the common and generic approach to RDE adopted by most of the world regions. It also includes the agreed specifications for the SPN PEMS equipment, which are already common to EU, Japan, South Korea, and China.

## 3. SPN-PEMS Evaluation 

This section will give the evolution of SPN-PEMS in chronological order. The evaluations started within the light-duty sector and then continued with the heavy-duty one.

### 3.1. Light-Duty

The light-duty PEMS evaluation followed three steps: (i) a theoretical study; (ii) an experimental evaluation in one laboratory with many vehicles and PEMS; (iii) an inter-laboratory correlation exercise at many laboratories with one vehicle and two PEMS. Table 1 gives an overview of the results of all studies. For DC-based systems, only results above 1 × 10^11^ p/km were considered in order to be well above their limit of detection. More details follow in the next sections.

#### 3.1.1. Theoretical Study

The call of interest for developing SPN-PEMS was announced in November 2012, and the kick-off meeting took place in April 2013. At that time, there were no commercially available SPN-PEMS. There was very limited experience with condensation particle counters (CPCs) for on-board measurements [19,20]. There were concerns though, regarding safety in the vehicle cabin due to the working fluid (butanol or propanol) and the effect of vibrations. 

Regarding the handheld CPCs (model 3007 and P-trak from TSI, Shoreview, MN, USA) [47,48], there were a few studies in the literature, which found differences within 20% for airborne particles between the handheld CPCs [49,50], or between the CPCs and a scanning mobility particle sizer (SMPS) when the appropriate size range was considered [51]. 

There was also some experience from the real-time detectors that were used for PM-PEMS. The most common ones were based on light absorption [52] and diffusion charging [53]. The light absorption detectors (Microsoot sensor, AVL, Graz, Austria) measure black carbon. Based on SPN-soot correlation studies, approximately 2 × 10^12^ particles correspond to 1 mg of soot [11]. However, the signal of the soot sensors (mass-based) is theoretically proportional to particle diameter to the power of 3, and typically for agglomerates (fractals) to the power of 2.3 [11,54].

The response of diffusion chargers (DCs) (surface area) is proportional to particle diameter squared for small particles. For the size range of interest (20–200 nm), an exponent of 1.1–1.3 to the particle mobility diameter is usually found. The exponent depends on the instrument characteristics but also on other parameters. For example, fractal particles acquire more charge than compact particles of the same mobility [55], and 10–30% higher charge can be measured for 100 nm particles. If particles are already charged with the same polarity of the charger, the final charging level can be up to 30% higher [56].

**Figure 1:**
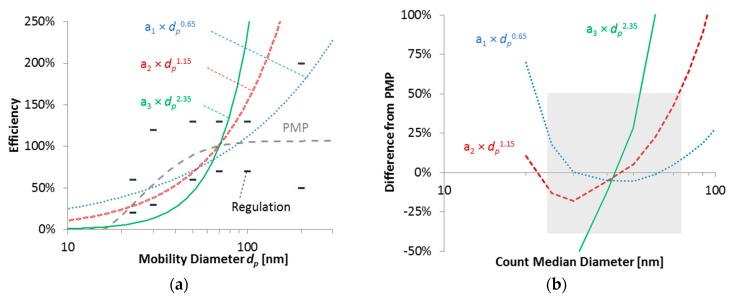
Theoretical study. Comparison of various SPN-PEMS to a PMP system. Adapted from [43]: (**a**) efficiency of systems in function of monodisperse mobility diameter *d_p_*; single points give the final efficiency requirements in the regulation; (**b**) differences of systems from the PMP system in function of the count median diameter of polydisperse aerosol. Shaded area covers acceptable differences. SPN = solid particle number; PEMS = portable emissions measurement system; PMP = particle measurement programme.

A theoretical study was conducted in order to assess whether DCs or light-absorption instruments could be an acceptable alternative to the CPC-based PMP reference systems [43]. Figure 1a plots a typical PMP counting efficiency curve (based on the technical requirements, see Appendix A) along with three theoretical systems that have a response to the power of 0.65, 1.15, and 2.35 in function of the monodisperse mobility diameter. The 2.35 simulates a light-absorption instrument, the 1.15 a diffusion charger, and the 0.65 an advanced instrument. Figure 1b plots the differences of the three systems from the PMP system for polydisperse aerosols with various count median diameters. Considering a range of −35% to +50% as an acceptable range of differences, it is clear that the light-absorbing instrument would often not correlate well with the PMP system, due to the high size dependency. The DC system (exponent 1.15) would have acceptable differences for count median diameters between 25 nm and 75 nm. The more advance instrument would have acceptable differences for a wider size range. At that time, it was assumed that a constant value determined at a specific monodisperse size (70 nm) would be adequate. However, as Figure 1b shows, this would not necessarily be optimum for all systems. For example, the system with exponent 0.65 could be optimised at a bigger size. Although the study showed that DCs could be an acceptable alternative to CPCs, the calibration procedure was left open. The first draft was based on the Swiss regulations for construction machinery (the final Swiss Regulation SR 941.242 was published in 2015). The final PEMS efficiency requirements were decided after the second campaign in 2014 (see below and Appendix A). Application examples were published in 2018 [57] after the introduction of SPN-PEMS with Regulation (EU) 2017/1154.

The results of the theoretical study were confirmed experimentally with some prototype SPN-PEMS (details about the instruments in Appendix B) using monodisperse and polydisperse spark-discharge graphite particles (DPN 3000 from Palas, Karlsruhe, Germany) [58] or thermally stable diffusion flame soot particles (APG from AVL, Graz, Austria) [59] (Table 1). The exponents for soot were 1.16 to 1.35 for the various DCs. The differences to the PMP systems for polydisperse aerosol were up to +97%. The results were also in agreement with those reported for handheld instruments used in workplaces for assessing personal exposure to airborne nanomaterials [60] or air monitoring studies [61]. 

#### 3.1.2. Experimental Evaluation in a Single Laboratory

**Figure 2:**
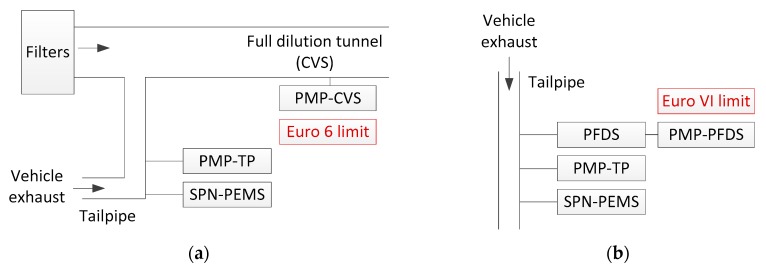
Typical experimental setup for the evaluation of solid particle number (SPN) portable emissions measurement systems (PEMS). (**a**) Light-duty; (**b**) heavy-duty. Reference systems according to the particle measurement programme (PMP). The SPN-PEMS systems were installed at the tailpipe of the vehicles and were compared with the reference system at the full dilution tunnel (PMP-CVS) or the proportional partial flow systems (PMP-PFDS), as required by the regulation. Comparisons with a PMP system at the tailpipe (PMP-TP) were also conducted in order to distinguish the effect of the ‘location’ from the SPN-PEMS instrument uncertainty.

The experimental evaluation was based on measurements in one laboratory (Joint Research Centre, JRC) with many vehicles and PEMS (Figure 2a), in two phases: Phase I, with DC-based instruments and using a constant to convert the signals of the instruments to particle number and no thermal pre-treatment requirements. Phase II, which included CPC-based instruments and more advanced DC-based instruments that could decrease the dependency on the size of particles and thermal pre-treatment similar to the PMP systems. In all cases, reference PMP systems were connected to the tailpipe and the full dilution tunnel with constant volume sampling (CVS). Note that the PMP at CVS is the system used to set Euro 6 limits for SPN. 

The first phase, at the end of 2013, evaluated DC-based prototype SPN-PEMS on a chassis dynamometer laboratory [44]. Three gasoline direct injection (GDIs) and one diesel particulate filter (DPF) equipped vehicles were tested. The best performing SPN-PEMS (DC-based, NanoMet 3 from Testo, Lenzkirch, Germany) was up to 83% when compared to the reference system at the dilution tunnel (CVS) at emission levels >1 × 10^11^ p/km (Table 1). This exercise showed that the differences between SPN-PEMS and PMP systems were higher than theoretically expected. Thus, new stricter SPN-PEMS efficiencies were recommended in order to reduce the measurement uncertainty of DCs at the PMP levels (Figure 1b, points). 

**Figure 3:**
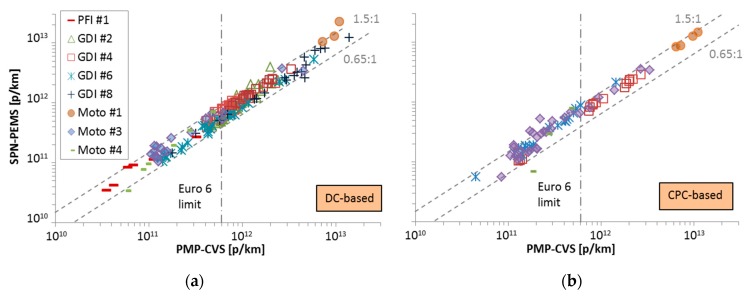
Joint Research Centre (JRC) phase II results. Adapted from [45]: Comparison of the best performing SPN-PEMS with the reference system at the full dilution tunnel (PMP-CVS). Each point is a phase of a test cycle. (**a**) Diffusion charger (DC) based; (**b**) condensation particle counter (CPC) based. GDI = gasoline direct injection; PFI = port-fuel injection.

At the second evaluation phase (September 2014 until the end of the same year), eight SPN-PEMS, five of them DC-based, were compared with legislation compliant SPN systems connected to the tailpipe and the dilution tunnel (CVS) [45]. The SPN-PEMS were calibrated by the instrument manufacturers. More than 130 tests were conducted with seven gasoline direct injection (GDI), three port fuel injection (PFI), two diesel particulate filter (DPF) equipped vehicles, one moped, and three motorcycles. The best performing SPN-PEMS (DC-based, the same as in Phase I, NanoMet 3 from Testo) was within +50% (with only a few exceptions) from the reference instrument at the dilution tunnel (Figure 3a and Table 1 as Testo (DC)). 

Two of the CPC-based SPN-PEMS had issues mainly with their dilution systems, which may be explained because they were prototype instruments. However, one CPC-based SPN-PEMS (modified NPET from Horiba, Kyoto, Japan) [62], which arrived late in the program, showed equivalent results with the reference systems (Figure 3b, and Table 1 as Horiba(CPC)). In addition, the real-time signal was correlating better with the reference systems compared to the DC-based systems. Further testing with another 10 vehicles after the campaign confirmed these findings [45]. Interestingly, the two best performing instruments were originally designed for the Swiss regulation for periodic inspection of off-road construction machinery [63].

#### 3.1.3. Inter-Laboratory Comparison Exercise

The two best performing systems, one DC- (Nanomet 3), and one CPC-based (modified NPET) were further assessed through an inter-laboratory comparison exercise (ILCE) [46]. The ILCE aimed to assess the accuracy and precision of the SPN-PEMS methodology using one vehicle in different laboratories across Europe directly involving other stakeholders, such as industry and technical services. The SPN-PEMS ILCE took place between September and December 2015. The participant labs were (alphabetically): Audi (Germany), BOSMAL Automotive R&D Institute (Poland), Honda Europe (Germany), TÜV Nord (Germany), Volvo (Sweden), VW (Germany). JRC started and closed the ILCE. One more laboratory (EMPA) measured in May 2016. The tests included regulated cycles in the laboratory and on-road RDE compliant tests. For the laboratory tests, a PMP system connected to the tailpipe was also circulated to all laboratories. 

**Figure 4:**
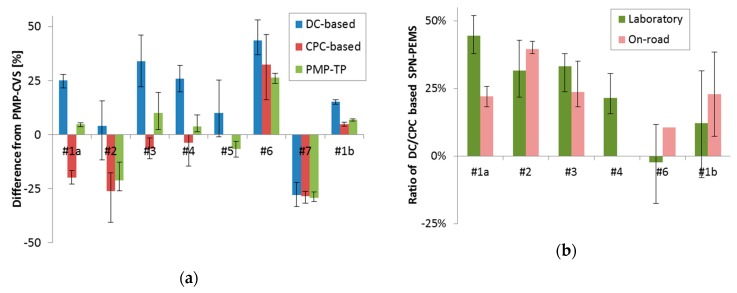
Light-duty inter-laboratory comparison exercise (ILCE) results. Adapted from [46]. (**a**) Deviation of the SPN-PEMS from the reference systems at the full dilution tunnel (PMP-CVS) at various European labs. The deviation of the reference system at the tailpipe is also given (PMP-TP). (**b**) Ratios of the DC- to CPC-based SPN-PEMS for the tests in the various laboratories and on-road tests. Error bars show min-max values of 2–5 tests, when available.

Both the DC- and CPC-based SPN-PEMS were within 50% of the PMP-CVS systems (Figure 4). However, it should be noted that a big contribution for these deviations could be attributed to some of the PMP-CVS systems that were over- or underestimating (i.e., they probably had calibration issues). This was evident from the similar scatter of the reference PMP system connected at the tailpipe (e.g., laboratories #2, #6, and #7 in Figure 4a).

For the on-road evaluation of the two SPN-PEMS (where there is no PMP-CVS or PMP-TP as a reference), their on-road ratio was compared to their laboratory ratio. Figure 4b presents the results. The same ratios were calculated for both on-road and laboratory tests, indicating that the performance of the SPN-PEMS did not change on the road.

#### 3.1.4. Measurement Uncertainty

Regulation (EU) 2017/1154, the third part of the RDE, introduced SPN-PEMS with a margin of 0.5 that takes into account the approximately 50% measurement uncertainty of SPN-PEMS. This margin was based on the two best-performing SPN-PEMS and (i) the experimental evaluation at JRC with many vehicles; (ii) the ILCE at many European laboratories with the two SPN-PEMS; (iii) a theoretical analysis of the measurement uncertainty based on the technical specifications. The theoretical analysis was similar to the first theoretical study (Figure 1) but considering the final technical specifications of SPN-PEMS. A maximum 30% difference was found between SPN-PEMS and PMP systems for the size range of interest (approximately 25–75 nm). An additional 20% uncertainty was considered for the differences between tailpipe and CVS (“location” effect). The emissions at the tailpipe can be higher because particle losses, mainly agglomeration, reduce the concentration until the CVS [30,45].

### 3.2. Heavy-Duty

At the end of September 2015, in a heavy-duty vehicles PEMS meeting, it was decided to evaluate the SPN-PEMS method due to its better sensitivity compared to the filter-based PM-PEMS method. The kick-off meeting for the SPN-PEMS for heavy-duty vehicles pilot study was held in Ispra (Italy) at the end of October 2015. Although SPN-PEMS had improved, for heavy-duty vehicles, wider conditions had to be investigated (e.g., compressed natural gas (CNG) vehicles, high content biofuels, regenerations, ambient temperatures −7 to +35 °C that were not tested at the light-duty studies, etc.). The evaluation started at JRC with Phase I and II, and then the engine manufacturers tested the SPN-PEMS (Table 2). Some further evaluations were done for DC-based instruments to understand some of their high deviations. For DC-based systems, only results above 1 × 10^11^ p/km were considered, in order to be well above their limit of detection.

#### 3.2.1. Experimental Evaluation in a Single Laboratory

**Figure 5:**
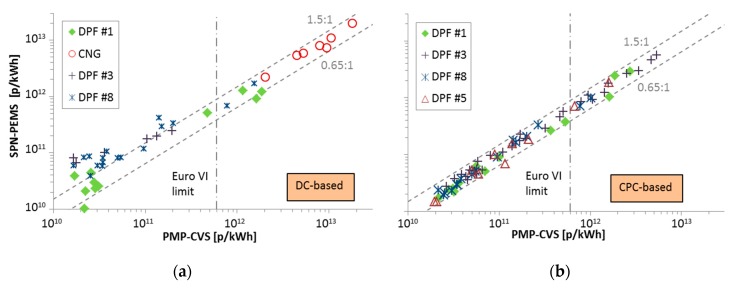
Heavy-duty JRC evaluation study on a chassis dynamometer (Phase I and Phase II). Adapted from [64,65]. Comparison of the best performing SPN-PEMS with the reference system at the full dilution tunnel (PMP-CVS). Each point is a test cycle. (**a**) Diffusion charger (DC) based; (**b**) condensation particle counter (CPC) based. CNG = compressed natural gas; DPF = diesel particulate filter.

Four SPN-PEMS instruments were evaluated on the heavy-duty chassis dynamometer of JRC and on the road from February until June 2016 [64]. In September 2016, the commercial instruments were tested [65]. The SPN-PEMS were based on the draft technical specifications of the light-duty RDE regulation. The differences of the SPN-PEMS to the reference system at the dilution tunnel (PMP-CVS) were found within 40% for temperatures >0 °C for both DC- (NanoMet 3 from Testo) (Figure 5a) and CPC-based (modified NPET from Horiba) (Figure 5b) systems. However, for the DC-based instrument, higher differences were found for concentrations 2 × 10^11^ p/kWh or lower, which was not seen at such degree in the light-duty study (Figure 3a).

#### 3.2.2. Inter-Laboratory Exercise

The testing took place in the facilities of European heavy-duty engines manufacturers from February 2017 until December 2017 (Table 2) [66]. The engine manufacturers were (alphabetically): CNH Industrial (Italy), DAF Trucks (Netherlands), Daimler AG (Germany), MAN Truck and Bus (Germany), Scania (Sweden), Volvo Powertrain Engineering (France). Each laboratory used at least one SPN-PEMS and a reference PMP system connected to a proportional partial flow dilution system (PFDS), as prescribed in the heavy-duty regulation. Many engines were tested (i.e., there was no “Golden” engine circulating). Two SPN-PEMS, one DC-based (Nanomet 3, Testo), and the OBS-ONE (the commercial version of the modified NPET from Horiba) were circulated to most of the laboratories. Mainly the type approval cycles with engine starting cold and/or hot were tested, but also steady cycles and regeneration events were conducted to challenge the systems with higher particle number concentrations and exhaust gas temperatures.

**Figure 6:**
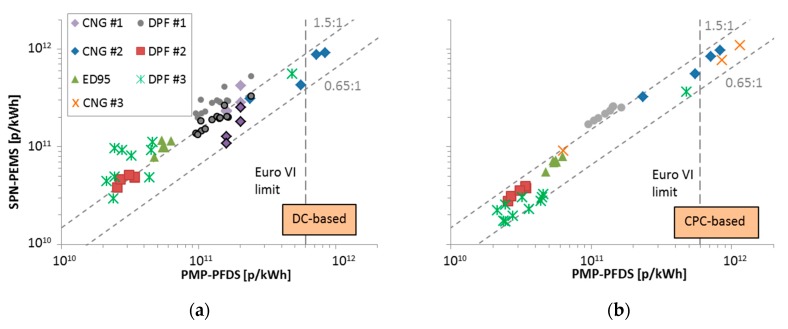
Heavy-duty inter-laboratory exercise (ILE) on engine dynamometers. Correlation between portable emissions measurement systems (PEMS) and reference particle measurement programme (PMP) systems for various engines. Adapted from [66]. (**a**) Diffusion charger (DC) based; (**b**) condensation particle counter (CPC) based. Symbols with a black frame line (CNG #1, DPF #1) for the DC-based system are results corrected with a PMP efficiency curve. DPF = diesel particulate filter; CNG = compressed natural gas; ED95 = 95% ethanol fuel.

Figure 6 compares the SPN-PEMS to the reference PMP systems connected to proportional partial flow dilution systems (PFDS). Each point is a test cycle. For the DC-based system (Figure 6a), the agreement was good, within −35% and +50%, but only for levels >3 × 10^11^ p/kWh. For lower levels the differences were much higher, reaching +165%. A significant reduction of the variance could be achieved by applying the PMP efficiency curve correction to the DC signal (based on the measured mean diameter by the DC): The points with emissions of 1–3 × 10^11^ p/kWh and the high differences could be brought within −35% and +50% (for the examined levels >1 × 10^11^ p/kWh) (see solid points with black frame in Figure 6a, details in [66]). For the CPC-based system (Figure 6b), there was good agreement for the whole examined range (10^10^–10^12^ p/kWh), with the exception of DPF #1, where differences around 70% were measured.

#### 3.2.3. Further Evaluations

The evaluations of SPN-PEMS with heavy-duty engine exhaust gave larger differences than what was observed for passenger cars (as discussed about Figure 5a vs. Figure 3a). In order to understand the differences, tests with two Euro VI technology diesel heavy-duty engines were conducted [68]. In agreement with other studies, it was found that urea injection leads to the formation of nonvolatile particles with a mean size below the regulatory limit of 23 nm [69,70]. The new finding was that these particles acquire high (more than one) positive charges at the exhaust temperatures exceeding approximately 300 °C [68]. The concentration of the sub-23 nm additional particles was in some cases more than twice of the 23 nm CPC concentration [68,71]. Consequently, small differences of the cut-off curves of the 23 nm CPCs can explain the high differences of Figure 6b. The high charge can explain the overestimation of emissions with the DC-based PEMS (Figure 6a). In the presence of positively charged particles <23 nm, the actual charge level of the aerosol exiting the corona is higher than anticipated for neutral particles, consequently the measured signal and the reported concentrations [67]. 

Two solutions were tested in the corona charger section of AVL’s SPN-PEMS [67]: Since a large fraction of the formed particles lies below the 23 nm detection threshold of the regulation, one straightforward solution was to remove all charged particles below this size via an electrofilter. A more advanced solution was to expose the pre-charged aerosol in a bipolar ion environment produced by a combination of a negative and a positive corona charger. Both technical solutions were found to efficiently address interferences from such particles, leading to deviations of less than 30% (from 120% at emission levels of 1 × 10^11^ #/kWh), with one exception (42%). 

#### 3.2.4. Measurement Uncertainty

Euro VI step E regulation was adopted in November 2019 and introduced SPN-PEMS from 2021 for compression ignition engines (2023 for positive ignition engines) with a conformity factor of 1.63 that takes into account the measurement uncertainty of SPN-PEMS. The 0.63 margin was based on the two best-performing SPN-PEMS and (i) the experimental evaluation at JRC with many vehicles; (ii) the engine manufacturers experimental assessment of many PEMS at many European laboratories; (iii) a theoretical analysis of the measurement uncertainty based on the technical specifications. The theoretical analysis was based on comparisons of PEMS with PMP systems fulfilling the upper and lower limits of their technical requirements. Additionally, particle losses that could take place at the PEMS or the PMP systems at the dilution tunnel were considered. Due to higher exhaust gas temperatures, the possible losses for heavy-duty vehicles were estimated to be higher than at light-duty vehicles (around 25%) [72]. In addition, due to the existence of small particles close to the cut-off size of the PMP systems, the differences of the PEMS and PMP systems were slightly higher (around 35%). The technical specifications and the measurement uncertainty of PEMS are under standardisation [73], further evaluation from metrological institutes [74], and subject to review in 2020.

## 4. Discussion

The first studies on SPN-PEMS started in 2013 [43], and comparisons with reference PMP systems were limited [28]. The evaluations were necessary in order to determine the measurement uncertainty that is introduced by the instruments. Today, PEMS are part of the European regulation [75]. In addition, many researchers use them for on-road studies [76,77,78]. As it is not possible to quantify the measurement uncertainty in every study, the previous analysis gave the expected range for various commercial instruments. The next paragraphs will discuss their robustness and the future studies.

### 4.1. Robustness

In general, the robustness and reliability of the equipment improved over time; however, robustness remains instrument specific. As none of the instruments were tested long enough to judge robustness (typically 3–5 months), the following issues that were encountered give indications of the challenges of current and future designs. It should be kept in mind that PMP systems also had issues of drift [79] and could also have issues when sampling directly from the tailpipe. Condensation of exhaust aerosol is probably the most critical challenge. The influence of the tubing has also been reported [29,80].

Most prototypes SPN-PEMS had issues such as malfunction of (prototype) detectors, failure of heated line, condensation at the electrometers, leakage between the sampling lines, drift of the diluter. These were addressed by the instrument manufacturers at their next versions. On the other hand, the Pegasor (DC) was the same unit at both light-duty evaluation phases without any maintenance in between and did not have any issues. The instrument was not further tested because it was not fulfilling the updated technical efficiency requirements. Recent dual concepts (i.e., two systems with different cut-off sizes by applying different trap voltage) need further evaluation in practice [81]. 

The AVL (DC) started with issues that were gradually solved. At the end of the light-duty evaluation, it had performance close to the best performing instruments. At the heavy-duty campaigns, some issues with low ambient temperatures were solved by adding probe heaters. The sensitivity at small charged particles formed by urea injection resulted in overestimation of the emissions. This was solved by the manufacturer implementing tandem chargers.

The Testo (DC) (NanoMet 3) was practically used at all measurement campaigns. In the light-duty ILCE, it showed 85% error-free tests on the dynamometer and 90% on-road. There were issues with condensation (CNG vehicle). The system was not evaluated at sub-0 °C ambient temperatures because it is designed for temperatures 5–35 °C.

The Horiba (CPC) (modified NPET) had 87% error-free tests on the dyno and 65% on-road (it was not available in one lab because it was at the instrument manufacturer for maintenance) at the light-duty ILCE. The commercial unit (OBS-ONE) did not show any issues during an Italian round robin with a gasoline car [82]. At the heavy-duty evaluation, the modified NPET from Horiba was not very robust at low ambient temperatures (<0 °C) when leaving it overnight at that temperature; most units tested failed after tests at low ambient temperatures. Further improvements regarding robustness against low ambient temperatures were introduced in the final product.

The Sensors (CPC) had issues at the beginning (light-duty evaluation) because it was a prototype. In one laboratory during the heavy-duty evaluation showed good results, but further studies are needed.

The Maha (CPC) was tested only a few times; nevertheless, it did not show any issues at any ambient temperature, but it was underestimating the emissions in both light-duty and heavy-duty studies.

### 4.2. The Future of SPN-PEMS

The SPN-PEMS technical specifications follow the laboratory PMP systems specifications. An important requirement is the 50% efficiency at approximately 23 nm. The European Commission is considering whether there is a need to lower the cut-off size from 23 nm to 10 nm [14]. Two issues are important in this consideration: Evidence that a high fraction of solid particles lie below 23 nm for some technologies, and whether the methodology for measuring below 23 nm can be reproducible and repeatable. The high percentage of solid particle reside in the sub-23 nm region was shown for both light-duty [75,83] and heavy-duty vehicles [71]. As the introduction of the particle number limit practically forced diesel particulate filter, the introduction of RDE practically forced gasoline particulate filters at gasoline direct injection engines. The lower cut-off size will probably force filters at other technologies if introduced in the future regulations. The SPN-PEMS specifications will have to follow this size reduction. New studies will, therefore, be necessary to confirm the comparability with the future reference PMP system and their measurement uncertainty.

Another big difference is that the technical specifications of the SPN-PEMS refer to the complete system, while those of the PMP system refer separately to the volatile particle remover (VPR) and the particle number counter (PNC). Although treating the SPN-PEMS as a “black box” gives more freedom to designs and technical solutions, it may result in higher measurement uncertainties. Partly, the measurement uncertainty could be reduced by further tightening the limits of the technical specifications. One difficulty that remains is the linearity check that needs high concentrations, and typically, a PMP system is used as a reference [57]. Another one is the calibration material: Soot for SPN-PEMS, open for PMP systems (typically emery oil for CPCs). Different calibration material can result in differences in the counting efficiencies [79]. Studies that will try to get the two procedures to converge will follow.

Tightening the efficiency specifications might mean that only CPCs will be possible as detectors or even more advance diffusion charging instruments will be necessary. For example, in the first theoretical study in 2014, the differential mobility spectrometer engine exhaust particle sizer (EEPS) from TSI was also used. The EEPS had already been used in a minivan with hot dilution in 2010 [84] and even earlier in 2007, the DMS50 from Cambustion (Cambridge, UK) [85]. The Electrical Low Pressure Impactor (ELPI) from Dekati (Tampere, Finland) has also been used on-board of heavy-duty vehicles [86]. Hand-held versions of differential mobility spectrometers are already available [87].

It should also be mentioned that part of the measurement uncertainty value used in the regulation comprises the particle losses between tailpipe (i.e., PEMS sampling location) and dilution tunnel (i.e., laboratory-grade equipment sampling location). There is an intention to permit SPN laboratory measurements for type approval also from the tailpipe [30,88]. This will further decrease the uncertainty from the different locations (at the moment estimated 20–25%).

In addition to PMP, there is and will be an interaction of SPN-PEMS with the specifications of the new periodical technical inspection (NPTI) for particle number equipment [89] and on-board monitoring (OBM) or on-board diagnostics (OBD) sensors [90,91].

## 5. Conclusions

The portable emissions measurement systems (PEMS) were introduced in the United States regulation more than a decade ago in order to check the compliance of heavy-duty engines in use without removing the engine from the vehicle. Europe followed in 2014 only for the gaseous pollutants of heavy-duty engines. A major change in the light-duty regulation was the real-driving emissions (RDE) regulation, which requires PEMS testing during type approval since 2017, not only for gaseous pollutants but also for solid particle number (SPN). The SPN-PEMS will also be used on heavy-duty vehicles in 2021. 

The European studies of SPN-PEMS underpinning the introduction of these systems in European legislation started in 2013 with prototype instruments. The first evaluations of diffusion-charger (DC) based system gave differences of around 100% from the reference laboratory systems measuring exhaust particles from light-duty vehicles. The technical requirements were tightened to be closer to the laboratory systems, and in 2014 the advanced DC-based systems and a CPC-based system were within 50% of the reference systems. An inter-laboratory comparison exercise in 2015 with a Golden vehicle and two Golden PEMS (one DC- and one CPC-based) confirmed the findings: The differences were within 50% from the participating laboratories systems. Testing with heavy-duty vehicles and engines in 2016 and 2017 revealed some issues at lower temperatures which were addressed at the commercial systems. One important topic was the higher variability with the DC-based systems even at levels of 35–50% of the particle number limit. Dedicated tests in 2018 showed that urea particles were charged at higher temperatures, resulting in overestimation of the DC systems signal. This issue was solved, bringing down the measurement uncertainty of DC systems to the expected levels of <50%. Today, PEMS are robust systems used for regulatory purposes. Their use is not restricted only to heavy-duty engines testing but also to light-duty vehicles. Their worldwide acceptance resulted in a Global Technical Regulation, which is under development. Most countries have introduced or will introduce them in future regulations. Further tightening of the regulatory technical requirements and/or improvements from the instrument manufacturers are needed to decrease the PEMS measurement uncertainty. At the same time, their use for research purposes is also increasing. The recent changes of the laboratory methodology to include particles <23 nm will result in modifications of the technical specifications, the regulations, and further PEMS studies.

## Figures and Tables

**Table 1:** Summary of light-duty studies. Min–max deviations from the reference system at the tailpipe PMP-CVS. For DC-based instruments values refer to emission levels >1 × 10^11^ p/km. Instrument details are in Appendix B.

SPN-PEMS	Exponent [43] (DC’s; Soot)	Theoretical ^1^ [43] Mid 2013	Phase I [44] Oct–Dec 2013	Phase II [45] Sep–Dec 2014	ILCE ^2^ [46] Sep–Dec 2015
AVL (DC)	1.26	−45% to +43%	−42% to +102%	−49% to +48%	−
Horiba (DC) ^3, 4^	1.16	−71% to +32%	−96% to +95%	+11% to +150%	−
Testo (DC)	1.18	−44% to +24%	−49% to +83%	−48% to +55%	−39% to +42%
Pegasor (DC) ^3^	1.35	−33% to +97%	−43% to +118%	−58% to +199%	−
Sensors (DC) ^4^	(1.35)	−44% to +90%	−35% to +188%	−85% to +309%	−
Shimadzu (DC) ^3^	−	−	−	−35% to +97%	−
Sensors (CPC)	−	−	−	issues	−
Horiba (CPC)	−	−	−	−21% to +49%	−41% to +54%
Maha (CPC)	−	−	−	−45% to +49%	−
PMP (CPC)	−	−	−29% to +56%	−28% to +49%	−

^1^ For count, median diameters 30 nm to 85 nm. ^2^ The scatter was less when PEMS were compared to the tailpipe PMP. ^3^ Not compliant with last regulated specifications. ^4^ Concept prototype (discontinued). CPC = condensation particle counter; DC = diffusion charger; ILCE = inter-laboratory comparison exercise; PEMS = portable emissions measurement system; PMP = particle measurement Programme; SPN = solid particle number.

**Table 2:** Summary of heavy-duty studies. Min–max deviations from the reference system at the proportional partial flow sampling system (PMP-PFDS). For DC-based instruments values refer to emission levels >1 × 10^11^ p/km. Instruments details are in Appendix B.

SPN-PEMS	Phase I [64] Feb–Jun 2016	Phase II [65] Sep 2016 ^1^	ILE [66] Feb–Dec 2017	Additional [67] Jan–Apr 2018
AVL (DC)	-	3% to +111%	42% ^1^	−24% to +42%
Testo (DC)	−39% to +40%	36% to +84%	−59% to +165%	−
Sensors (CPC)	-	-	−44% to −14% ^1^	−
Horiba (CPC)	−43% to +32%	−9% to +25%	−37% to +79%	+2% to +38%
Maha (CPC)	-	−47% to −18%	−	−
PMP (CPC)	−22% to +34%	−13% to +5%	−33% to 46% ^1^	−

^1^ Based on one engine.CPC = condensation particle counter; DC = diffusion charger; ILE = inter−laboratory exercise; PEMS = portable emissions measurement system; PMP = particle measurement programme; SPN = solid particle number.

**Figure A1:**
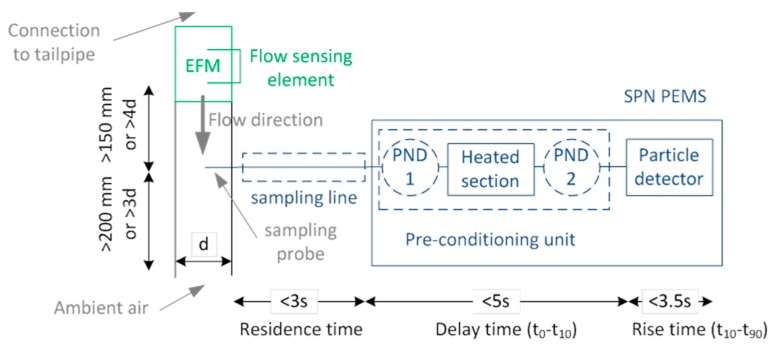
Example of an SPN-PEMS setup: Dotted lines depict optional parts. Adapted from Regulation (EU) 2017/1154. EFM = exhaust mass flow meter; d = inner diameter; PND = Particle Number Diluter.

## Appendix A

This Appendix describes the basic technical requirements of the PMP systems and the SPN-PEMS.

**PMP:** The basic technical requirements of the PMP methodology as implemented in the European regulation were: It shall consist of a volatile particle remover (VPR), which removes volatile particles and dilutes the sample, and a Particle Number Counter (PNC) that counts the particles >23 nm. The VPR shall dilute the sample at least 10 times at temperature ≥150 °C and have an evaporation tube at temperature 350 °C. The particle number concentration reduction factor (PCRF) at 30 and 50 nm must be lower than 1.3 and 1.2 times the PCRF at 100 nm, respectively, where PCRF is the ratio of upstream to downstream concentrations of monodisperse particles of the specific size. The PNC, practically a CPC, shall be full flow (no internal splitting) with a response time of <5 s and counting efficiencies of 0.5 ± 0.12 and >0.9 at 23 and 41 nm, respectively. The slope, as determined by comparing with a reference instrument (e.g., electrometer) at the whole measurement range, shall be 1 ± 0.1, with differences of each point (except the zero) within 10% of the reference.

The system shall achieve >99% removal of ≥30 nm tetracontane (CH_3_(CH_2_)_38_CH_3_) particles with an inlet concentration of ≥10.000 p/cm^3^ at the minimum dilution.

**SPN-PEMS:** The most important technical specifications of SPN-PEMS (called particle number analyser in the Regulation (EU) 2017/1154 are described below (Figure A1).

If particles are not diluted at the tailpipe, the sampling line shall be heated to a minimum temperature of 100 °C until the point of first dilution of the SPN-PEMS or the particle detector of the PEMS. The residence time in the sampling line shall be less than 3 s. The delay time of the SPN-PEMS shall be ≤5 s. The SPN-PEMS (and/or particle detector) shall have a rise time of ≤3.5 s. All parts in contact with the sampled exhaust gas shall always be kept at a temperature that avoids condensation of any compound in the device. The SPN-PEMS shall include a heated section at wall temperature ≥300 °C.

The complete SPN-PEMS, including the sampling line, shall fulfil the efficiency requirements of Figure 1a. Efficiency at a size *d_p_* is defined as the ratio in the readings of the SPN-PEMS to a reference CPC’s (50% counting efficiency at 10 nm or lower, checked for linearity and calibrated with an electrometer) or an electrometer’s number concentration measuring in parallel monodisperse aerosol of mobility diameter *d_p_* and normalised at the same temperature and pressure conditions. The material should be thermally stable soot-like (e.g., spark discharged graphite or diffusion flame soot with thermal pre-treatment).

The SPN-PEMS, including the sampling line, shall fulfil the linearity requirements of slope 1.00 ± 0.15, offset <5% of max concentration, R^2^ > 0.95 using monodisperse or polydisperse soot-like particles. The particle size (mobility diameter or count median diameter) should be larger than 45 nm. The reference instrument shall be an electrometer or a CPC with 50% counting efficiency at 10 nm or lower, verified for linearity, or a PMP system. In addition, the differences of the SPN-PEMS from the reference instrument at all points checked (except the zero point) shall be within 15% of their mean value. At least 5 points equally distributed (plus the zero) shall be checked. The maximum checked concentration shall be the maximum allowed concentration of the SPN-PEMS.

The system shall achieve >99% removal of ≥30 nm tetracontane (CH_3_(CH_2_)_38_CH_3_) particles with an inlet concentration of ≥10.000 p/cm^3^ at the minimum dilution. The system shall also achieve a >99% removal efficiency of polydisperse alcane (decane or higher) or emery oil with count median diameter >50 nm and mass >1 mg/m^3^.

## Appendix B

This Appendix describes the basic characteristics of the systems that were used during the European SPN-PEMS studies.

*AVL (PMP):* The APC 489 from AVL (Graz, Austria) was used in most labs and measurement campaigns [92]. It consisted of a hot dilution at 150 °C (25:1), an evaporation tube at 350 °C and a secondary dilution at ambient temperature (10:1), followed by a condensation particle counter (CPC) (model 3790 from TSI, MN, USA) with 50% counting efficiency at 23 nm [93].

*Testo (PMP):* The Nanomet 1 from Testo (Lenzkirch, Germany) (formerly Matter Engineering) consisted of an MD19-2E rotating disc diluter [94] followed by an ASET15-1 thermodiluter. The sample was diluted at the sample point with the rotating disc diluter using conditioned air at 150 °C. The diluted sample was then thermally treated at 350 °C in an evaporation tube and subsequently diluted in a simple air mixer diluter at a rate of 10:1. A PCRF of 150–550 was employed in most tests. A CPC 3790 from TSI having a 50% efficiency at 23 nm was connected. This system was used mainly at the tailpipe.

*Horiba (DC) (prototype, discontinued):* The prototype instrument from Horiba (Kyoto, Japan) consisted of a VPR and a DC (DCS-100 from TSI). The VPR consisted of a heated ejector diluter (180 °C), a heated line (47 °C) that brought the diluted sample to a secondary ejector diluter at ambient temperature. The DC gave ‘aerosol length’ in mm/cm^3^ [95], which was converted to p/cm^3^ by multiplying with a constant. The specific DC was used with diffusion screens to reduce the sensitivity to sub 23 nm particles.

*Pegasor (DC):* The PPS-M from Pegasor (Tampere, Finland) consisted of a VPR and DC at the same unit [96,97]. The sample was drawn through a 2 m heated line by an ejector pump. The sample line and the whole unit with the DC were heated at 200 °C. The DC was based on the electrical detection of an aerosol following the “escaping current” technique. A sample of the exhaust gas was charged by a corona-ionised flow as it was being pumped by an ejector diluter built in the sensor’s construction. While the majority of the corona ions returned to the grounded sensor’s body due to their high electrical mobility, a small quantity was lost with the charged particles exiting the sensor. This “escaping current” was a measurement of the particle concentration in the exhaust gas.

*Sensors (DC) (prototype, discontinued):* The prototype form Sensors (Saline, MI, USA) consisted of a VPR and a DC (PPS from Pegasor). The VPR consisted of a primary diluter and a heated line at 47 °C. The DC primarily measured the escaping current, which was converted to particle number concentration with an internal constant.

*AVL (DC):* The particle number module of MOVE from AVL (Graz, Austria) consisted of a short (0.9 m) heated line at 150 °C with a sampling rate of 0.7 l/min, a 2:1 hot dilution at 150 °C, an evaporation tube and a catalytic stripper (both set at 300 °C), and a secondary dilution 3:1 at 60 °C. A 1.3 m heated line at 60 °C connected the secondary diluter to the particle detector (modified Partector, from Naneos, Windisch, Switzerland) [98]. In the detector, particles were firstly charged in a corona charger, then a pulsed electric field periodically removed a fraction of charged particles, and finally, the rate of change of the aerosol space charge in a Faraday cage was measured contactless (aerosol measurement with induced currents) [98].

*Horiba (CPC):* The particle number module of OBS-ONE from Horiba (Kyoto, Japan) was a modified version of the NanoParticle Emission Tester (NPET) (from TSI, MN, USA) [62]. The first diluter (10:1) was located directly at the sample probe at the tailpipe. A 2.5 m heated line at 60 °C, a heated catalytic stripper at 350 °C, and a second dilution (10:1) followed. The detector was an isopropyl alcohol-based CPC with 50% efficiency approximately at 23 nm (a TSI CPC 3007 with modified saturator and condenser temperatures) [47].

*Testo (DC):* The NanoMet 3 from Testo (Lenzkirch, Germany) (formerly Matter Engineering) had a 3 m heated line at 115 °C to transfer the exhaust in the main unit. The main unit consisted of a rotating disc diluter, an evaporation tube at 300 °C, and a mini Diffusion Size Classifier (DiSC mini, Testo) [99]. The DiSC mini consisted of a unipolar diffusion charger, a diffusion stage where the smallest particles were deposited by diffusion, and a filter stage where the remaining particles ended up. With the ratio and sum of the two currents, an average particle diameter and particle concentration were estimated. By applying a correction to the measured concentration based on the PMP efficiency (Figure 1a) of the DiSC estimated average particle diameter, a better estimate of the SPN concentration >23 nm could be achieved (e.g., see Figure 6a points with frame).

*Sensors (CPC):* The condensation particle number (CPN) module from Sensors (Saline, MI, USA) consisted of a sampling probe with a heated line at 100 °C, a hot diluter at >150 °C (dilution 30:1), a catalytic stripper (350 °C), a second stage diluter 150:1, and a CPC from Sensors.

*Shimadzu (DC):* The Nano Aerosol Monitor (NAM) from Shimadzu (Kyoto, Japan) was a six-stage electromobility analyser without sheath flow [100] downstream of a heated line at 190 °C and a diluter (10:1). The cut-off size was 23 nm. The particle concentration was estimated by the difference of the last and first electrometers, multiplied with the dilution and the range calibration factor.
